# Comprehensive Analysis of Photosynthetic Characteristics and Quality Improvement of Purple Cabbage under Different Combinations of Monochromatic Light

**DOI:** 10.3389/fpls.2016.01788

**Published:** 2016-11-29

**Authors:** Biyun Yang, Xiangzhu Zhou, Ru Xu, Jin Wang, Yizhang Lin, Jie Pang, Shuang Wu, Fenglin Zhong

**Affiliations:** ^1^College of Horticulture, Fujian Agriculture and Forestry UniversityFuzhou, China; ^2^Institute of Vegetable Crops, Fujian Agriculture and Forestry UniversityFuzhou, China; ^3^College of Food Science, Fujian Agriculture and Forestry UniversityFuzhou, China; ^4^Haixia Institute of Science and Technology, Fujian Agriculture and Forestry UniversityFuzhou, China

**Keywords:** purple cabbage, lighting conditions, LED, photosynthetic characteristics, vegetative quality

## Abstract

Light is essential for plant growth. Light intensity, photoperiod, and light quality all affect plant morphology and physiology. Compared to light intensity, photoperiod, little is known about the effects of different monochromatic lights on crop species. To investigate how different lighting conditions influence crops with heterogeneous colors in leaves, we examined photosynthetic characteristics and quality (regarding edibility and nutrition) of purple cabbage under different combinations of lights. Eight different treatments were applied including monochromic red (R), monochromic blue (B), monochromic yellow (Y), monochromic green (G), and the combination of red and blue (3/1, RB), red/blue/yellow (3/1/1, RBY), red/blue/green (3/1/1,RBG), and white light as the control. Our results indicate that RBY (3/1/1) treatment promotes the PSII activity of purple cabbage, resulting in improved light energy utilization. By contrast, both G and Y lights alone have inhibitory effect on the PSII activity of purple cabbage. In addition, RBY (3/1/1) significantly boosts the anthocyanin and flavonoids content compared with other treatments. Although we detected highest soluble protein and vitamin C content under B treatment (increased by 30.0 and 14.3% compared with the control, respectively), RBY (3/1/1) appeared to be the second-best lighting condition (with soluble protein and vitamin C content increased by 8.6 and 4.1%, respectively compared with the control). Thus we prove that the addition of yellow light to the traditional combination of red/blue lighting conditions is beneficial to synthesizing photosynthetic pigments and enables superior outcome of purple cabbage growth. Our results indicate that the growth and nutritional quality of purple cabbage are greatly enhanced under RBY (3/1/1) light, and suggest that strategical management of lighting conditions holds promise in maximizing the economic efficiency of plant production and food quality of vegetables grown in controlled environments.

## Introduction

Light is one of the most important environment regulators for the growth of crop species. It provides essential energy input and triggers various signaling pathways for dynamic growth regulation of crops. Classically, the light refers to white light, which is a mixture of a wide range of different wavelengths (colors). Previous research already discovered the differential responses of plant morphology and physiology to specific spectrum of light (Hogewoning et al., 2010). It is now well known that Red light (R) and blue light (B) can be more effectively absorbed by photosynthetic pigments (Pfundel and Baake, 1990). Thus, the combination of R and B has been widely used as light resource under controlled cultivation conditions. However, there is controversy about the optimal ratio between the R and B in different backgrounds. For example, it was reported that plantlets of strawberry and rapeseed, as well as cucumber seedlings gained highest fresh and dry weight with 7/3 ratio of B/R (Nhut et al., 2003), while other research showed that the optimal ratio is 1/3 (Li et al., 2013) and 9 (Hernandez and Kubota, 2016).

More studies have been performed to dissect the role of each individual wavelength of light. However, neither monochromatic R nor B alone appeared to be sufficient for maintaining plant growth. Reduced photosynthetic rate (Pn) or even aberrant leaf morphology were observed under R or B alone in many plant species (Wang et al., 2009; Hogewoning et al., 2010). But interestingly, Su et al. (2014) had found that the maximal photochemical efficiency of PSII (Fv/Fm) and the photosynthetic rate were all increased in cucumber seedlings grown under blue light as compared with those grown under white light. Therefore, the requirement for optimal dose of each light wavelength may depends on individual species.

**FIGURE 1:**
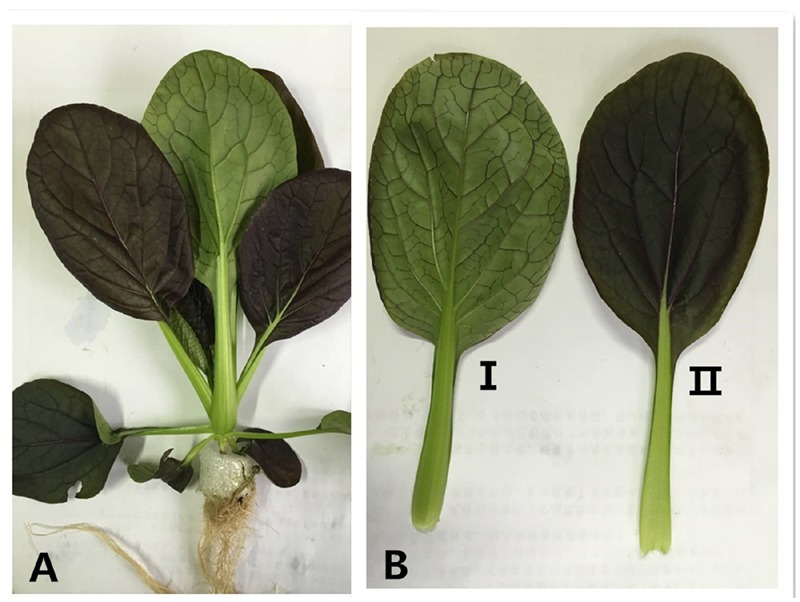
**A seedling of Purple cabbage grown in liquid culture (A**: the whole seedling of the Purple cabbage; **B-I**: abaxial side of Purple cabbage leaf; **B-II**: adaxial side of Purple cabbage leaf).

As the light sensing organ, most crops produce leaves in uniformly green. To investigate how the colority of leaves could affect the response to lighting conditions, we studied a variation of Chinese cabbage, Purple cabbage (*B. campestris* ssp. *chinensis* var. *communis* Tsen et Lee), which has purple leaf surface on adaxis side and green leaf surface on abaxis side (**Figure 1**). This allows the dissection of the effect of different ratio between anthocyanin and chlorophyll on photosynthesis under different combinations of monochromic light.

In this paper, we utilized a cultivated species purple cabbage ‘ziwei’, and performed a comprehensive analysis of photosynthetic characteristics including PSII activity, biomass, accumulations of chlorophyll (Chl) and carotenoids (car), as well as vegetable quality traits such as content of soluble sugar, soluble protein and vitamin C under different combinations of monochromatic lighting conditions. Our results indicated that the mixture light of red / blue / yellow (3/1/1) was the best to increase efficiency for solar energy utilization, pigment contents and other qualities of purple cabbage. Our findings can help to determine appropriate light settings for purple cabbage cultivation, and provide a theoretical and practical basis for further study on effect of different monochromic lights on plant growth.

## Materials and Methods

### Materials and Designs

The experiments were performed at Vegetable Research Institute, Fujian Agriculture and Forestry University on November 2013. The purple cabbage (ziwei) was purchased from Beijing Jingyu Wei’er Agricultural Technology co., Ltd. Seeds were sterilized with warm water and put into potted trays (The volume of the pots is 0.0664 m^3^) which contained 7.5 kg (V(garden soil):V(turf soil):V(perlite) = 5:7:3 and included 20% organic matter).

Nutrient liquid was applied every five days and the ingredient was based on a modified protocol adapted from Lycoskoufis with an electrical conductivity (EC) of 1.1 dS m^-1^ and pondus hydrogen ii (pH) of 5.7. (Lycoskoufis et al., 2005). The nutrient composition was as follows: 4.74 mM K^+^, 2.0 mM Ca^2+^, 1.0 mM Mg^2+^, 0.74 mM P^5+^, 1.0 mM NH_4_^+^, 7.0 mM NO_3_^-^, 2.1 mM SO_4_^2-^, 0.74mM H_2_PO_4_^-^, 46 μM B, 92 μM Fe, 9.6 μM Mn, 0.77 μM Zn, 0.32 μM Cu, 0.016 μM Mo. Other treatments were performed as usual. During the four leaves period, the new bud with the same growth condition were used for light treatments. The EC value of the nutrient solution was measured by DDS-12A conductance instrument (Shanghai Hongyi Instrumentation co., Ltd). The temperature was set to 25°C and DJS - 1 was selected as conductivity electrode. The measurement was performed after standard calibration. The calculation was based on the formula of Sonneveld (0.095^∗^9.63+0.19 = 1.1), in which 9.63 corresponds to the sum of the cations in meq.

Eight treatments were include in our study: White light (CK, 100% white light), Red light (R, 100% red light), Blue light (B, 100% blue light), Yellow light (Y, 100% yellow light), Green light (G, 100% green light), the mixture of Red light and Blue light (RB, R/B = 3:1, 75% red light plus 25% blue light), the mixture of Red light, Blue light and Yellow light (RBY, R/B/Y = 3:1:1, 60% red light, 20% blue light plus 20% yellow light), the mixture of Red light, Blue light and Green light (RBG, R/B/G = 3:1:1, R/B/G = 3:1:1, 60% red light, 20% blue light plus 20% green light). LED light source was the integration of tubes which is produced by Shenzhen Vanq Technology co., Ltd. Each tube contained 20 led bulls whose power is 20 w. We mixed Red (660-670 nm; absorption peak at 660 nm), blue (440-445 nm; absorption peak at 435 nm), yellow (660-670 nm; absorption peak at 590 nm) and green (515-530 nm; absorption peak at 520 nm) according to different proportion by average arrangement of light quality lamp bead as a control, we used white light which has 8990k color temperature and 471.7 nm dominant wavelength. To test each light source, we used the system of Yuanfan PMS-50 SSA_V 1 to test the light source, and measured the photosynthetic photon quanta flux density and light density by GLZ-C Photosynthetic effective radiometer from Tuopu Company, Zhejiang, China. The frame was made of steel and the inside was covered with aluminum coated reflective film. The whole structure was covered with black shading cloth outside. The light source was on the top of the culture frame, with 15 ± 5 cm distance from the plants. This setup allows the photosynthetic photon quanta flux density kept at 100 ± 5 μmol/m^2^/s. Ten pots for each group, and three replicates for each treatment. The cycle was from (8:00-20:00), 12 h/d for 15 days. The daytime temperature was kept at 25 ± 1°C, and dark temperature at 15 ± 1°C.

### Determined Indexes and Methods

#### Photosynthetic Rate

In the morning, the No.4 and No.5 leaves under the top of plant were chosen for experiments. Photosynthetic rate, stomatal conductance, transpiration rate, and Intercellular CO_2_ concentration were measured by CI-340 Portable photosynthesis system.

#### Chlorophyll Fluorescence

After 20 min’s adaption to dark conditions, the N0.4 and No.5 leaves under the top of plant were chosen for fluorescence parameter analysis by Handy PEA from England. All experiments were repeated at least three times. **Table 1** showed the index of fluorometric determination.

**Table 1:** Formulas of chlorophyll fluorescence parameters.

Chlorophyll fluorescence parameters	Formulas
ABS/RC	=M_O_^∗^(1/V_J_)^∗^(1/ψ_PO_)
TR_O_/RC	=M_O_^∗^(1/V_J_)
ET_O_/RC	=M_O_^∗^(1/V_J_)^∗^ψ_O_
DI_O_/RC	=(ABS/RC)-(TR_O_/RC)
PI_ABS_	=RC/ABS[ψ_PO_/(1-ψ_PO_)][Ψ_O_/(1-Ψ_O_)]
ψ_PO_	=TR_O_/ABS=F_V_/F_M_
Ψ_O_	=ET_O_/TR_O_=(1-V_J_)
ψ_EO_	=ET_O_/ABS=(1–F_O_/F_M_)Ψ_O_
M_O_	=4(F_k_-F_O_)/(F_M_-F_O_)
W_k_	=(F_k_-F_O_)/(F_J_-F_O_)
V_J_	=(F_J_-F_O_)/(F_M_-F_O_)

#### Anthocyanin Measurement

The extraction of anthocyanin was performed according to Ghafoor et al. (2011). 0.5 g of leaves (dry mass, DW) was submerged in 8 mL of a solution containing 0.05% hydrochloric acid and 40% ethanol with ultrasonic assisted extraction by KQ-600DE. After centrifugation at 4000 × *g* for 10 min, the anthocyanin content was measured spectrophotometrically at 530 and 700 nm. The formula follows Wrolstad et al. (2005):

$$\begin{array}{c} \text{Total}\;\text{Anthocyanins}\;(\text{mg/L})\;\text{=}\;(\text{A}\times \text{MW}\times \text{DF}\times {\text{10}}^{\text{3}})\text{/}(\varepsilon \times \;\text{l})\\ \begin{array}{c} \begin{array}{c} \text{A}\;\text{=}\;(\text{A}\;\text{max-A700}\;\text{nm})\;\text{pH}\;\text{1}.\text{0-}(\text{A}\;\text{max-A700}\;\text{nm})\;\text{pH}\;\text{4}.\text{5 }\\ \text{MW}\;\text{=}\;\text{Molecular}\;\text{Weight} \end{array}\\ \text{DF}\;\text{=}\;\text{Dilution}\;\text{Factor} \end{array}\\ \varepsilon \;\text{=}\;\text{molar}\;\text{extinction}\;\text{coefficient},\;\text{L}\times {\text{mol}}^{\text{-1}}\times {\text{cm}}^{\text{-1}}\\ \text{ l}\;\text{=}\;\text{pathlength}\;(\text{1}\;\text{cm}) \end{array}$$

#### Flavonoids Measurement

The determination of flavonoids content with ultrasonic assisted extraction was performed according to Zhou et al. (2011). Portulaca flavonoids extraction method was improved as: With 60% ethanol concentration, solid-liquid ratio was 1:25, ultrasonic temperature was 35°C, ultrasonic time was 30 min, ultrasonic power was 70 W. The content was determined by sodium nitrite-aluminum nitrate, sodium hydroxide method.

#### Soluble Protein Measurement

Soluble proteins were measured according to Salcedo et al. (2010). One gram of leaves (fresh mass, FW) were ground up in a mortar with liquid nitrogen, to which 25 mL distilled water. The extract was centrifuged at 13,000 × *g* for 10 min, and 1 mL of the supernatant was mixed with 5 mL Folin-A (which was composed by 10 g Na_2_CO_3_, 2 g NaOH, and 0.25 g KNaC_4_H_4_O_6_⋅4H_2_O into 500 mL volume flask) and 0.5 mL Folin-B (which take 0.5 g CuSO_4_⋅4H_2_O dilute with distilled water to 500 mL volume flask. After 30 min, the nitrate content was measured at a wavelength of 650 nm.

#### Soluble Sugar Measurement

Soluble sugar were measured by Hernandez and Hernandez (1994). 0.2 gram of leaves (fresh mass, FW) was submerged in 10 mL (V) of distilled water. After 30 min in a water bath at 85°C, the supernatant was collected. 0.5 milliliter of the supernatant was combined with 1.5 mL of distilled water. 0.5 mL sulfuric acid anthrone and 5 mL concentrated sulfuric acid were added. After shaking 1 min, the soluble sugar content was determined with the sulfuric acid anthrone method at a wavelength of 630 nm.

#### Vitamin C Measurement

Vitamin C were measured by Rasaki et al. (2008). 2 g of leaves samples (fresh mass, FW) were mixed with 3 mL 2% oxalic acid. After shaking, added 1 mL 30% zinc sulfate and 1 mL 15% potassium ferrocyanide. The supernatants were used to determine the concentration of Vitamin C. The vitamin C content was determined with the 2, 6-dichlorophenol indophenol sodium staining method at a wavelength of 500 nm.

#### Photosynthetic Pigments Measurement

Photosynthetic pigments were measured by Solovchenko et al. (2011). 0.2 gram of leaves (fresh mass, FW) was submerged in 10 mL (V) of 80% acetone. The pigment was extracted until the leaf turned white. Optical density (OD) was measured with a TU-1810 spectrophotometer at 470 nm for carotenoid (OD 470 nm), at 663 nm for chlorophyll a (OD 663 nm), and at 645 nm for chlorophyll b (OD 646 nm), and calculated by the following equations as follow.

$$\begin{array}{c} \text{Chl}\;\text{a}\;(\text{mg}\cdot {\text{g}}^{\text{-1}})\;\text{=}\;(\text{12}.\text{21}\;\text{OD663-2}.\text{81}\;\text{OD646})\text{V/1},\text{000}\;\text{W}\\ \text{Chl}\;\text{b}\;(\text{mg}\cdot {\text{g}}^{\text{-1}})\;\text{=}\;(\text{20}.\text{13}\;\text{OD646-5}.\text{03}\;\text{OD663})\text{V/1},\text{000}\;\text{W}\\ \text{Carotenoid}\;(\text{mg}\cdot {\text{g}}^{\text{-1}})\;\text{=}\;\frac{(\text{1},\text{000}\;\text{OD470-3}.\text{27}\;\text{Chl}\;\text{a}\;\text{-}\;\text{104}\;\text{Chl}\;\text{b})\;\text{V}}{\left(\text{229}\times \text{1},\text{000}\;\text{W}\right)} \end{array}$$

where *V* is the total volume of acetone extract (mL), and *W* is the fresh weight (*g*) of the sample

### Data Collection and Statistical Analysis

All measurements were replicated three times, and the experiment was repeated twice to check the reproducibility of results with 10 plants in each treatment. The data were analyzed by one-way analysis of variance (ANOVA) and the differences between the means were tested using LSD’s multiple range test by DPS(V3.01) (*P* < 0.05).

## Results

### The Influence of Different Monochromic Lights on Photosynthetic Parameters of Purple Cabbage

**FIGURE 2:**
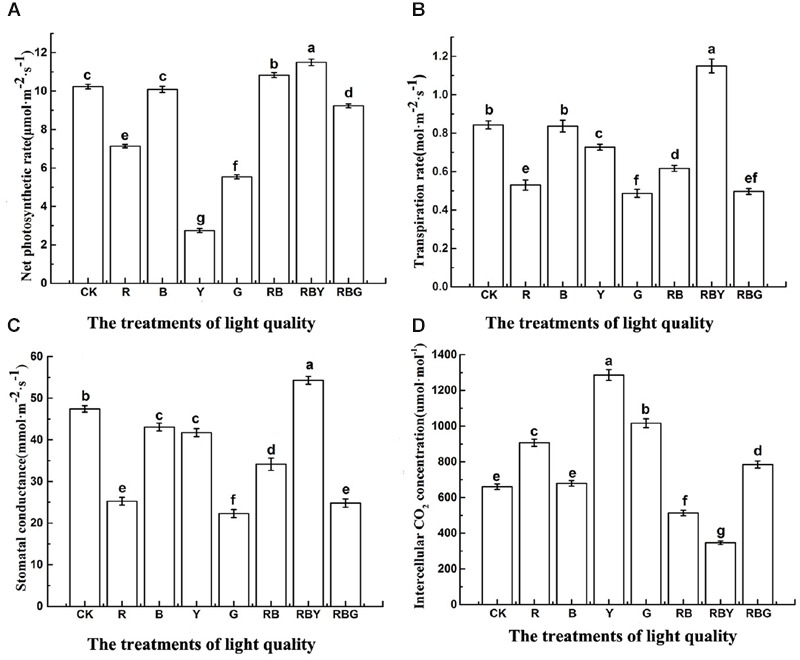
**The effects of light treatment on Pn (A)**, Tr **(B)**, Gs **(C)**, Ci **(D)** of purple cabbage, White light (CK), Red light (R), Blue light (B), Yellow light (Y), Green light (G). Values were the means of three replicates with standard errors shown by vertical bars. Different letters indicate significant differences using the LSD’s Multiple Range Test (*p* < 0.05, *n* = 3).

As showed in **Figure 2**, different monochromic light settings led to substantially different photosynthetic parameters of purple cabbage. In terms of Pn, the effect on photosynthetic parameter of purple cabbage was RBY > RB > CK > B > RBG > R > G > Y. Under RBY treatment, the value of photosynthetic parameters were significantly higher than other treatments, with the value of 12.3% higher than CK. RB treatment was in the second place which showed 5.8% higher than CK. However, Y treatment resulted in the lowest Pn values (2.7 μmol⋅m^-2^⋅s^-1^), which was 73.2% reduction compared with CK treatment. As for Ci value, the effect of different light combination was Y > G > R > RBG > B > CK > RB > RBY. Two other commonly used photosynthetic parameters, Gs and Tr exhibited the same tendency as: RBY > CK > B > Y > RB > R > RBG > > G. Under RBY treatment, both values showed the highest level with 36.4 and 14.5% increase, respectively, compared to CK treatment. However, Ci and Pn value gave opposite trend, with Y showing the highest value and RBY showing the lowest level.

### The Influence of Different Monochromic Lights on Chlorophyll Fluorescence Parameters of Purple Cabbage

**FIGURE 3:**
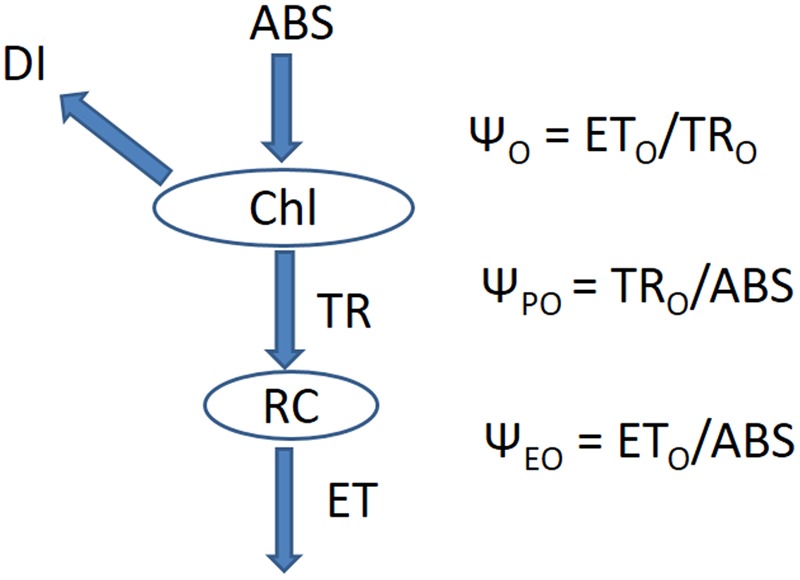
**The energy cascade from light absorption to electron transport.** Chl indicates chlorophyll, ABS indicates the absorption of energy, DI indicates the heat dissipation of energy, RC indicates the active reaction centers in the photosynthetic system, TR indicates the energy captured by the active reaction centers, ET indicates the energy for electron transport.

To understand whether effect of different light combinations arise from chlorophyll content and function, we further measured a number of parameters associated with chlorophyll fluorescence. The relationships among all parameters are listed in **Figure 3**.

**FIGURE 4:**
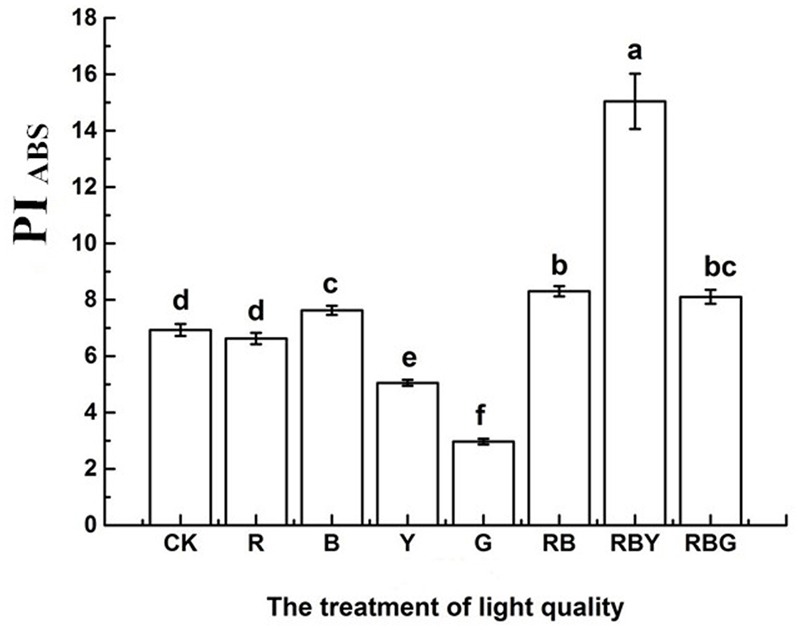
**The effects of lighting conditions on photosynthetic PI_ABS_ of purple cabbage.** White light (CK), Red light (R), Blue light (B), Yellow light (Y), Green light (G). Values were the means of three replicates with standard errors shown by vertical bars. Different letters indicate significant differences using the LSD’s Multiple Range Test (*p* < 0.05, *n* = 3). PI_ABS_ indicates the performance index on absorption basis.

As showed in **Figure 4**, RBY combination gave rise to the highest value of PI_ABS_ of purple cabbage and the order of different light mixtures were: RBY > RB > RBG > B > CK > R > Y > G. The value of RBY treatment was 1.2 times higher than that of CK treatment. Meanwhile, G treatment was the lowest value among all treatments, being 57.1% lower than CK treatment. These data proved that after being treated by different light combinations, photosynthetic process in purple cabbage exhibited dramatic changes. Among all treatments, RBY treatment gave the best photosynthetic performance, which is helpful for absorbing light and transferring it to stable chemical energy.

**FIGURE 5:**
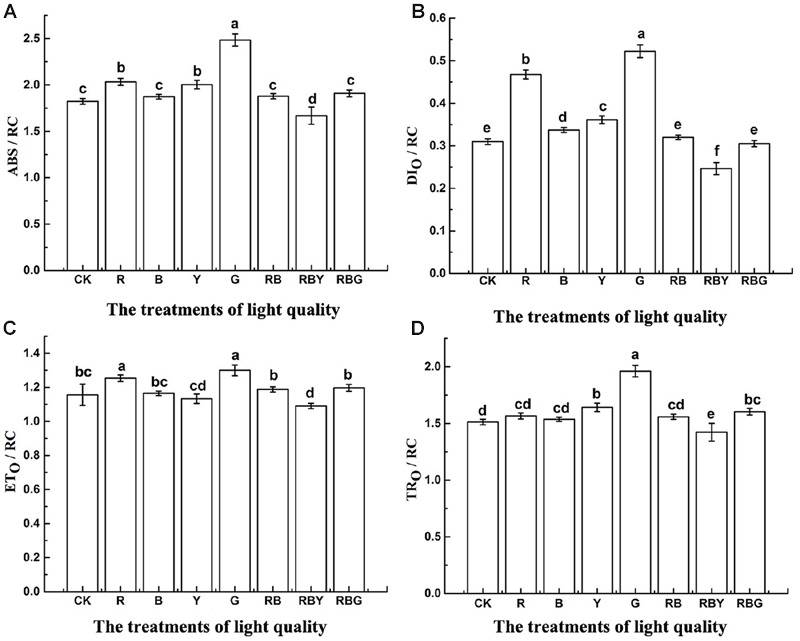
**The effects of lighting conditions on ABS/RC (A)**, DI_O_/RC **(B)**, ET_O_/RC **(C)**, TR_O_/RC **(D)** of purple cabbage. White light (CK), Red light (R), Blue light (B), Yellow light (Y), Green light (G). Values were the means of three replicates with standard errors shown by vertical bars. Different letters indicate significant differences using the LSD’s Multiple Range Test (*p* < 0.05, *n* = 3). ABS/RC, DI_O_/RC, ET_O_/RC, TR_O_/RC indicate absorption rate, energy capture rate, electron transport rate and dissipated energy per active reaction centers, respectively.

In addition to PI_ABS_, some other parameters that are based on the reaction of the active center, were often used to describe the chlorophyll fluorescence and function. As shown in **Figure 5**, opposite to that in PI_ABS_, all values of ABS/RC, TR_O_/RC, ET_O_/RC, DI_O_/RC reached the highest level under the G treatment, but dropped to the lowest level under RBY treatment. G treatment increased the energy of ABS/RC, TR_O_/RC, and ET_O_/RC (with 36.3, 29.7, and 12.4% increase, respectively, compared with the control), which represents light absorption and energy transfer. However, G treatment also showed the highest level of DI_O_/RC (with 68.6% increase compared with the control), which reflects the heat dissipation of energy. That prevents the transfer of absorbed energy to the electronic chain end, resulting in the inhibition of PSII activity. Therefore, the gross light energy assimilation of G light was lower than the other light treatments. The value of DI_O_/RC of all treatments exhibited the trend as: G > R > Y > B > CK > RB > RBG > RBY, suggesting monochromic light has lower light energy assimilation than the light combinations in purple cabbage.

**FIGURE 6:**
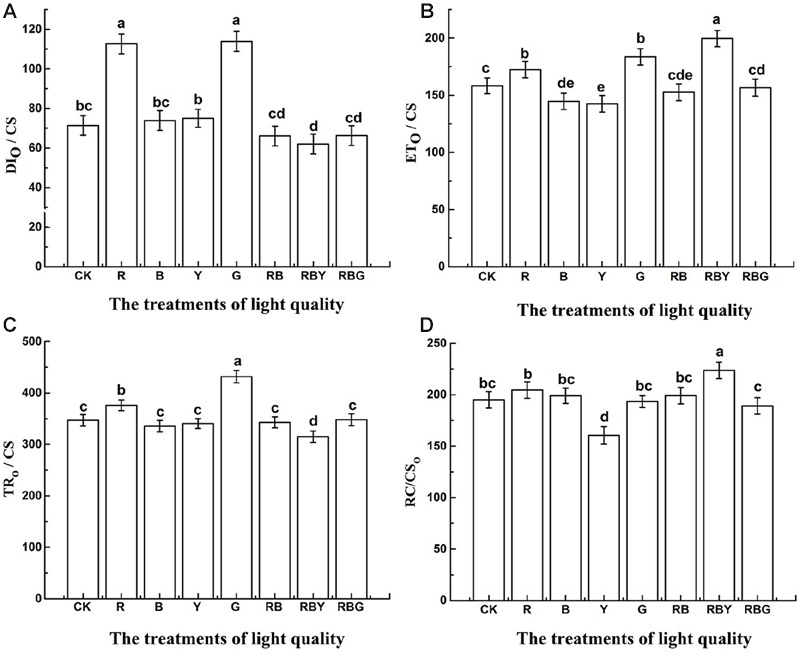
**The effects of lighting conditions on DI_O_/CS (A)**, ET_O_/CS **(B)**, TR_O_/CS **(C)**, RC/CS_O_ **(D)** of purple cabbage. White light (CK), Red light (R), Blue light (B), Yellow light (Y), Green light (G). Values were the means of three replicates with standard errors shown by vertical bars. Different letters indicate significant differences using the LSD’s Multiple Range Test (*p* < 0.05, *n* = 3). TR_O_/CS, ET_O_/CS, RC/CS_O_, DI_O_/CS indicate energy capture rate, electron transport rate, dissipation and density of PSII reaction centers per excited cross-section respectively.

To further understand how photosynthesis efficiency is affected under different light combinations, we measured the value of energy flow per unit area (CS). As showed in **Figure 6**, the values of TR_O_/CS, DI_O_/CS, which represents energy transfer and heat dissipation per unit area, reached the lowest level under the red, blue and yellow light combinations, and were 9.2 and 13.1% lower than CK treatment, respectively. In line with this, ET_O_/CS and RC/CS_O_, which represent energy transfer and the density of PS II reaction centers per unit area, showed the highest value under RBY treatment, and were 26.1%, 14.7% higher than CK treatment. Therefore, light combinations enhanced the efficiency of light energy utilization compared to monochromic light. Under G treatment, TR_O_/CS and DI_O_/CS were higher (with 24.4 and 59.6% increase, respectively, compared with the control), while RC/CS_O_ were significantly lower (with decrease 0.8% compared with the control). These results indicate that the structure of PSII reaction center was more stable under RBY treatment.

**FIGURE 7:**
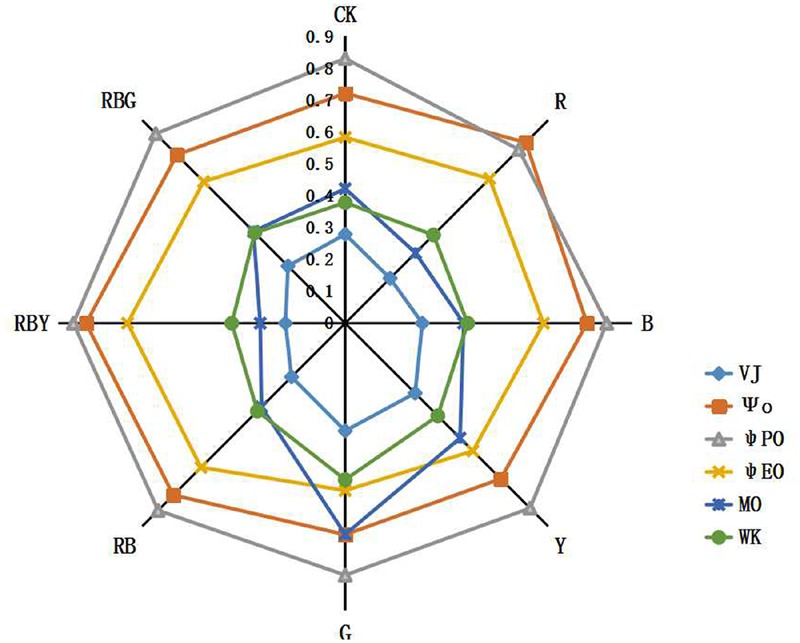
**The effects of lighting conditions on quantum yield parameters of purple cabbage.** White light (CK), Red light (R), Blue light (B), Yellow light (Y), Green light (G). ψ_PO_ (equivalent to F_V_/F_M_) represents the maximum quantum yield of PSII. Ψ_O_ represents the efficiency that a trapped exciton can move an electron to the downstream of Q_A_^-^ on the electron transport chain. ψ_EO_ represents the probability that an absorbed photon moves an electron to the downstream of Q_A_^-^ on the electron transport chain. V_J_ represents the relative variable fluorescence at J-step ration of fluorescence F_k_ to the amplitude F_J_-F_O_. MO represents the maximum rate of the reduction of Q_A_ and can be calculated as approximated initial slope of the fluorescence transient. W_k_ represents the ration of fluorescence F_k_ to the amplitude F_J_-F_O_.

The ultimate product of photosynthesis is the fixed carbon and the efficiency of it can be reflected by the quantum yield value. ψ_PO_ (equivalent to F_V_/F_M_) represents the maximum quantum yield of PSII, reflecting the energy capture efficiency of the reaction center Ψ_O_ represents the efficiency that a trapped exciton can move an electron to the downstream of Q_A_^-^ on the electron transport chain. ψ_EO_ represents the probability that an absorbed photon moves an electron to the downstream of Q_A_^-^ on the electron transport chain. MO, VJ, Ψ_O_, and ψ_EO_ mainly reflect changes in the electron transfer rate of the PSII receptor. The decline of those values suggests that a treatment inhibits the activity of either donor side or receptor side of the photosynthetic electron transport chain. These parameters are independent of each other and reflect the effect of different lights on the photosynthetic electron transport chain. In **Figure 7**, we showed that the values of ψ_PO_, Ψ_O_ and ψ_EO_ were significantly different under different light treatments. RBY combination had 2.7, 12.7, 17.1 higher ψ_PO_, Ψ_O_, and ψ_EO_, respectively, compared to the CK treatment. ψ_PO_ value under R treatment reached the lowest, which was 7.2% lower than CK treatment. However, the lowest value of Ψ_O_ and ψ_EO_ was detected under G treatment, which was 8.1 and 10.0% lower than that of CK treatment, respectively.

In addition, the PSII electron donor side represented by W_k_ and a receptor performance parameters represented by V_J_ also exhibited difference after different treatments. Compared to CK treatment, RBY treatment had 5.9 and 32.9% lower W_k_ and V_J_, respectively. Oppositely, values of W_k_ and V_J_ were significantly increased under Y and G treatment compared to the CK treatment, with W_k_ increased by 8.6% (Y) and 29.7% (G), and V_J_ increased by 11.2% (Y) and 20.0% (G).

The maximum rate of the reduction of Q_A_ can be calculated as approximated initial slope of the fluorescence transient, which is called M_O._ The maximum reducing rate of M_O_ represented by Q_A_ and reached the lowest value under RBY treatment, which was 36.8% lower than the control. Q_A_ reached maximum under G treatment which were 56.9% higher than CK treatment respectively. It indicated that after RBY treatment, the activity of PSII reaction center was increased.

### The Influence of Different Monochromic Lights on Photosynthetic Pigment Content of Purple Cabbage

We further ask whether the altered parameters shown in **Table 2** was partially caused by the change of photosynthetic pigment content. We next examined four different traits of photosynthetic pigment content, including chlorophyll a, chlorophyll b, chlorophyll (a + b), and chlorophyll a/b. As shown in **Table 2**, after different light treatments, all four values had changes in the purple cabbage leaves. Specifically, chlorophyll a, chlorophyll b and chlorophyll (a + b) reached the peak after R treatment. Compared with CK treatment, chlorophyll a and b content increased by 19.4 and 51.6%, respectively, and chlorophyll (a + b) increased by 26.4%. By contrast, chlorophyll a and chlorophyll b became the lowest after G and Y treatment, suggesting that G and Y treatments were inhibitory to the accumulation of photosynthetic pigments.

**Table 2:** Effect of different lights on vegetable quality of purple cabbage.

Light treatment	Anthocyanin (mg⋅g^-1^DW)	Flavonoids (mg⋅g^-1^FW)	Soluble protein (mg⋅g^-1^FW)	Vitamin C (mg⋅g^-1^FW)	Soluble sugar (mg⋅g^-1^FW)
CK	6.52 ± 0.07cC	3.95 ± 0.02bB	12.56 ± 0.11 dD	0.37 ± 0.01 cdCD	0.27 ± 0.02 cC
R	4.59 ± 0.09fF	3.67 ± 0.02cC	11.67 ± 0.13 eE	0.34 ± 0.02 eE	0.39 ± 0.01 aA
B	6.92 ± 0.07bB	4.04 ± 0.04bAB	16.33 ± 0.08 aA	0.42 ± 0.01 aA	0.24 ± 0.01 dD
Y	5.31 ± 0.08eE	3.25 ± 0.04eE	8.53 ± 0.11 gG	0.37 ± 0.01 cdCD	0.27 ± 0.01 cC
G	4.42 ± 0.07gG	3.51 ± 0.06dD	10.39 ± 0.12 fF	0.35 ± 0.02 deDE	0.08 ± 0.01 fF
RB	5.67 ± 0.07dD	4.04 ± 0.13bB	13.34 ± 0.13 cC	0.40 ± 0.01 bAB	0.24 ± 0.01 dD
RBY	7.18 ± 0.03aA	4.18 ± 0.02aA	13.65 ± 0.13 bB	0.38 ± 0.01 bcBC	0.32 ± 0.01 bB
RBG	5.76 ± 0.06dD	3.76 ± 0.04cC	10.29 ± 0.13 fF	0.37 ± 0.01 cCD	0.17 ± 0.01 eE

*White light (CK), Red light (R), Blue light (B), Yellow light (Y), Green light (G). Different letters indicate significant differences using the LSD’s Multiple Range Test (*p* < 0.05, *n* = 3).*

Different from the chlorophyll, carotenoid content of purple cabbages under B treatment was 29.0% higher than CK treatment, which was significantly higher than any other treatments. Other treatments such as RBY only elevated the carotenoid content of purple cabbages by 13.3% compared to CK treatment. Above results indicated that blue light is beneficial for increasing the content of carotenoid.

### The Influence of Different Monochromic Lights on Vegetable Quality of Purple Cabbage

Purple cabbage is rich in anthocyanin, which is antioxidant pigment and has a great potential of application in food industry, medicine and cosmetics. Thus we examined the effect of different monochromic lights on anthocyanin content in purple cabbage. As seen from **Table 2**, purple cabbage’s anthocyanin and flavonoids content reached the maximum under RBY treatment. Compared with CK treatment, anthocyanin and flavonoids content increased 10.1 and 5.8%, respectively. On the contrary, Y treatment appeared to be inhibitory and anthocyanin and flavonoids content were 18.6 and 17.8% lower than those of CK treatment, respectively. These results indicated that blue and yellow light are detrimental to the synthesis of flower flavonoids.

In addition, we examined a number of other factors that decide the vegetable quality of purple cabbage. As shown in **Table 2**, soluble protein and vitamin C values reached the peak after B treatment, which increased by 30.0 and 14.3% compared with CK treatment, respectively. RBY (3/1/1) appeared to be the second-best lighting condition with 8.6 and 4.1% increase, respectively compared with the control. However, Y treatment significantly reduced the value to 31.1%. Together, our results suggested that blue light was beneficial for production of soluble protein and vitamin C in purple cabbage. Differently, after the R treatment, the content of soluble sugar appeared to be significantly increased, which resulted in 42.4% increase compared to CK treatment. It indicated that R treatment was favorable to increasing the content of soluble sugar in purple cabbage.

## Discussion

Photosynthetic rate is the parameter reflecting the ability of plants to use light, to fix CO_2_ and to produce photosynthetic product. However, photosynthetic rate of plants can be considerably different in response to different monochromic lights. It was reported previously by Su et al. (2014) that cucumber seedlings exhibited a maximal photosynthetic rate under blue light. However, in our study, we found photosynthetic rate (Pn) value of purple cabbage under B treatment was lower than CK treatment. Interestingly, Pn under yellow and green light exhibited even more significant decline. Similar phenomenon was also observed by Korbee et al. (2005) in which red alga porphyras exhibited a minimum photosynthetic rate and electron transfer efficiency under blue light.

In addition, studies in lettuce leaves reported that green light can drive leaf photosynthesis more efficiently than red light and blue lights (Muneer et al., 2014; Golovatskaya and Karnachuk, 2015). In our study, under green light, Pn, Gs, PI_ABS_, ψ_PO_, Ψ_O_, and ψ_EO_ all were the lowest, while TR_O_/CS was the highest, which indicated under the green light processing, the ability of capture and transfer electron to Q_A_^-^ were to be reduced, the active center were closing on purple cabbage. That suggested that green light was not an ideal light condition for light use efficiency and can be detrimental to the PS II put oxygen complex optical system and its receptor side. Our results suggest that green light treatment hinders plant growth in purple cabbage, which was consistent with a number of observations in other species. (Folta and Maruhnich, 2007; Terashima et al., 2009). Although in our study, we detected the similar Pn values between yellow and blue light, ψ_PO_ value and the stomatal conductance under yellow light were significantly higher than those under the green light treatment. Therefore, Y treatment appeared to be more effective than G treatment for the photosynthesis of purple cabbage.

Traditionally, red and blue light mixture (RB, 3/1) was widely used as artificial lighting during the cultivation. Here, we found the addition of yellow light can greatly enhance the photosynthesis performance of purple cabbage. Under RBY (3/1/1) treatment, TR_O_/CS and DI_O_/CS were lower, while ET_O_/CS, RC/CS_O_, Ψ_O_,ψ_EO_, and PI_ABS_ was significantly higher than other treatments. This indicated that light energy conversion reaction center number was higher and PSII reaction center structure was comparatively stable under RBY treatment. But interestingly, M_O_ achieved the lowest level under RBY treatment which might be because the Q_A_ increased significantly. Another possibility is Q_A_ could markedly enhance its capability of electronic receptors for downstream reactions, and thus PSII reaction center receptor Q_A_ for electronic can quickly passed to the downstream side electron acceptor such as Q_B_ and PQ, without accumulation of Q_A_^-^ or Q_A_^2-^. As a result, this promoted the active state of PSII reaction center, and this possibility was also supported by previous results from Falqueto et al. (2010).

Light quality under controlled cultivation conditions can change growth, fresh weight and vegetable qualities of many horticultural crops, and thus can greatly affect their market value. Therefore, the study of LED lighting effect on vegetable growth and development emerged to be a hot topic in the field of vegetable cultivation (Bian et al., 2015). In our study, we set up four monochromatic lights and four combinations to explore their effect on purple cabbage traits. In our study, we detected higher Chl and Car content under red than under blue light. One possibility for this is that red light is more efficient in the induction of Chl and Car synthesis in leaves than blue light. Therefore, the red light appeared to be beneficial to pigment accumulation and secondary metabolite consumption. However, RBY (3:1:1) mixture exhibited the different trend from red light alone, suggesting the existence of interplay between different lights.

Many previous studies have reported that anthocyanin biosynthesis is an important process that depends on light. Our results showed that the content of anthocyanin and flavonoids was both increased under RBY treatment. Although the increase of anthocyanin can also be detected under blue light alone, RBY treatment achieved the highest level of anthocyanin and flavonoids. It suggested that compound light effect on the regulation of plant may be was not just a simple mixture of the role of monochromatic light but a more complex synergistic process. This may be the result of the interaction between light spectrum and plant’s own pigment system. It indicated that light quality balance is essential for normal plant growth (Piovene et al., 2015).

In addition to pigments, we detected the increase of soluble protein and vitamin C under the red light, blue light and RB mixture. However, treatment by yellow light alone caused the lowest concentration of soluble protein. Therefore, we speculated that the concentration and activity of soluble proteins could increase with the blue-ray spectrum that has relatively higher photon energy. In accordance with our findings, Qian et al. (2016) reported the increase of vitamin C and anthocyanin in Chinese kale sprouts under blue light treatment. In plants, galactonolactone dehydrogenase (GLDH) can directly catalyze the conversion of galactose ester into vitamin C. There was evidence suggesting blue light enhances the activity of GLDH, thus promoting the accumulation of vitamin C (Hodges and Forney, 2003).

In our study, we found soluble sugar showed the highest level under red light, while lowest level under green light. One possibility is that red light activated the phytochrome, which promotes the activities of sugar metabolic enzymes. This is also supported by previous research (Kasperbauer, 2000). Under green light, however, the sheet-like structure of chloroplasts could be disrupted and thus impaired the photosynthesis efficiency. That could lead to the lowest soluble sugar level with green light treatment.

Compared to individual monochromic light, the RBY combination effectively improved the light energy utilization and the photosynthetic pigment content, which eventually result in promoted nutritional qualities of purple cabbage. Our results provide the testable and tractable combination of the LED lights, and can facilitate the production of high-quality purple cabbage.

## Author Contributions

Conceived and designed the experiments: BY, FZ, SW. Performed the experiments: FZ, BY, SW, RX, JW. Analyzed the data: FZ, XZ, SW. Contributed reagents/materials/analysis tools: FZ, XZ, RX, YL, JP. Wrote the paper: BY, FZ, SW.

## Conflict of Interest Statement

The authors declare that the research was conducted in the absence of any commercial or financial relationships that could be construed as a potential conflict of interest.

## Abbreviations

ψ_EO_: Probability that an absorbed photon will move an electron into the electron to the downstream of Q_A_^-^ on the electron transport chain
Ψ_O_: Efficiency that a trapped exciton can move an electron to the downstream of Q_A_^-^ on the electron transport chain
ψ_PO_: (F_V_/F_M_) Maximum quantum yield of PSII
ABS/RC: Absorption per active reaction centers
B: monochromic blue LED light
Ci: intercellular CO_2_ concentration
CK: the white LED light
DI_O_/CS: Dissipation per excited cross-section
DI_O_/RC: Dissipated energy flux per active reaction center
DW: Dry weight
ET_O_/CS: Electron transport per excited cross-section
ET_O_/RC: Electron transport per active reaction center
F_M_: Dark-adapted maximum fluorescence
Fo: Dark-adapted minimum fluorescence
FW: Fresh weight
G: monochromic green LED light
Gs: stomatal conductance
M_O_: Approximated initial slope of the fluorescence transient
PI_ABS_: Performance index on absorption basis
Pn: net photosynthetic rate
Q_A_: Primary quinine accepter
Q_B_: Secondary quinine accepter
R: monochromic red LED light
RB: the combination of red and blue (3/1) LED light
RBG: the combination of red and blue and green (3/1/1) LED light
RBY: the combination of red and blue and yellow (3/1/1) LED light
RC/CS_O_: Density of PSII1reaction centers per excited cross-section
Tr: transpiration rate
TR_O_/CS: Trapping per excited cross-section
TR_O_/RC: Trapping per active reaction centers
V_J_: Relative variable fluorescence at J-step Ration of fluorescence F_k_ to the amplitude F_J_-F_O_
W_k_: Ration of fluorescence F_k_ to the amplitude F_J_-F_O_
Y: monochromic yellow LED light.
